# Synthesis, structure, Hirshfeld surface analysis, and molecular docking studies of the cocrystal between the Cu(II) complex of salicylic acid and uncoordinated piracetam

**DOI:** 10.55730/1300-0527.3700

**Published:** 2024-10-18

**Authors:** Nazokat N. YULDASHEVA, Ikram I. ABDULLAEV, Oybek I. KHUDOYBERGANOV, Lola A. GANDJAEVA, Pirnazar K. KODAMBOEV, Elyor Sh. SAMANDAROV, Adkhamjon S. NORMAMATOV, Abror Kh. RUZMETOV, Yuldosh Y. YAKUBOV, C. BALAKRISHNAN, Bakhtiyar T. IBRAGIMOV, Aziz B. IBRAGIMOV

**Affiliations:** 1 Khorezm Mamun Academy, Uzbekistan Academy of Sciences, Khiva, Uzbekistan; 2 Department of Biology, Urgench State University, Urgench, Uzbekistan; 3 Supramolecular Compounds Division, Institute of General and Inorganic Chemistry, Uzbekistan Academy of Sciences, Tashkent, Uzbekistan; 4 Department of Chemistry, Erode Sengunthar Engineering College, Perundurai, Erode, India; 5 Institute of Bioorganic Chemistry, Uzbekistan Academy of Sciences, Tashkent, Uzbekistan

**Keywords:** Salicylic acid, piracetam, Cu(II) complex, quantum-mechanical calculations, biological activity

## Abstract

The cocrystal (or supramolecular complex) between the Cu(II) complex of salicylic acid and uncoordinated piracetam has been synthesized. Its structure is characterized by elemental analysis, FT-IR, UV-Vis spectroscopy, and X-ray crystallography. Spectroscopic methods confirm the formation of the metal complex, while X-ray crystallography establishes the molecular and crystal structure of the obtained compound. The Cu(II) complex of salicylic acid (complex molecule) is a symmetric binuclear compound in the form of a “Chinese lantern” and contains 4 salicylic acid and 2 water molecules. It interacts with uncoordinated piracetam through a complicated system of hydrogen bonds. However, according to Hirshfeld surface analysis, the contribution of the O•••H/H•••O contacts is only 24.9%, while H•••H and H•••C/C•••H contacts account for 67.5%, indicating that intermolecular interactions are mainly hydrophobic. In silico (molecular docking) studies of the cocrystal, the complex molecule, and piracetam’s antifungal, antibacterial, and antiviral activities confirm that the complex molecule demonstrates enhanced biological activities; practically, the inactive piracetam improved all tested types of bioactivities through cocrystal formation. For example, the binding energy in the case of anti-COVID activity is improved from −10.34 to −11.40 kcal/mol. Thus, cocrystal formation based on metal complexes and inactive organic compounds may be promising in drug design.

## 1. Introduction

Cocrystals, formed between an active pharmaceutical ingredient (API) and a cocrystal former (or conformer), are attracting increasing interest within the pharmaceutical community as promising alternatives for solid drug formation. To date, scientists have synthesized various types of uncommon cocrystals containing metal complexes as crystal formers and APIs [1–3]. These cocrystals offer enhancements to various pharmaceutically relevant properties compared to single-component crystals, including improvement of solubility, dissolution rate, hydration stability, fluorescence performance, and bioavailability [4]. The interaction between the API and the cocrystal former occurs via nonionic and noncovalent intermolecular interactions, such as van der Waals forces and hydrogen bonding. Therefore, the presence of unused hydrogen bond donor and acceptor sites is crucial for cocrystal formation [5,6].

Salicylic acid is utilized across various fields, including agriculture, cosmetics, and emerging pharmaceuticals, due to its diverse biological properties. It is widely recognized as a crucial signaling molecule in regulating plant responses to environmental stress [7–13]. A literature survey reveals that numerous metal complexes formed with APIs have demonstrated greater efficacy in treating diseases compared to the original ligands alone [14,15].

Piracetam, also known as 2-oxo-pyrrolidineacetamide, is a nootropic drug widely used in the treatment of age-related cognitive decline and various nervous system disorders such as Alzheimer’s disease and dementia [16–18]. In the case of Cu(II) salts, salicylic acid tends to form binuclear tetrasalicylate metal complexes [19]. Due to the four uncoordinated hydroxy groups of the complex molecule, it can easily form cocrystals with APIs, such as piracetam. With its dual amide moieties, piracetam provides an appropriate model of a pharmaceutical compound for exploring cocrystal formation. It is of great interest to estimate the biological action of the complex molecule with salicylic acid, its cocrystal with piracetam, and piracetam itself. In this work, we synthesized a cocrystal based on salicylic acid and piracetam, determined its structure, and compared its antimicrobial and antiviral activities using an in silico (molecular docking) approach.

## 2. Materials and methods

All the used chemicals were obtained from Sigma-Aldrich (St. Louis, MO, USA) and used as received. The elemental percent compositions of compounds were determined with an elemental analyzer (CHNS/O, Unicube, Elementar, Jeddah, Saudi Arabia) using the Dumas method. Fourier transform infrared (FTIR) spectra were recorded on an IRTracer-100 FTIR spectrophotometer (Shimadzu, Tokyo, Japan) in the range of 4000–400 cm^−1^ with recording accuracy of 1 cm^−1^. Spectral data were processed using LabSolution IR software (Shimadzu). Electronic transitions in the compound were investigated using UV spectrophotometry (Cary 5000 UV-Vis-NIR, Agilent Technologies, Santa Clara, CA, USA) in the wavelength range of 200–1100 nm.

### 2.1. X-ray crystallography

Reflection sets for X-ray diffraction experiments were obtained at 293 K on an XtaLAB Synergy HyPix3000 diffractometer (Rigaku, Tokyo, Japan; microfocus sealed X-ray tube, single source at home/near, PhotonJet (Cu (λ = 1.54184 Å), X-ray source mirror monochromator, detector resolution of 100,000 pixels mm^−1^, *ω-*scans). Experimental data were collected using the CrysAlisPro program [20]. Absorption correction was applied by the multiscan method using the same program. The structure was solved by direct method using the SHELXT program package [21] and refined by full-matrix least squares using the SHELXL program [22]. All nonhydrogen atoms were refined anisotropically. Molecular drawings were plotted with the MERCURY program package [23]. The crystallographic data and details of the structural refinement are given in Table 1. Crystallographic data were deposited in the Cambridge Crystallographic Data Centre (Deposition Number: 2344454).

### 2.2. Synthesis

2-Hydroxybenzoic acid (0.276 g, 2 mmol) was dissolved completely in 30 mL of ethanol by stirring. Subsequently, a solution of 0.142 g (1 mmol) of 2-oxo-1-pyrrolidine acetamide in 10 mL of ethanol was poured into the previous solution and thoroughly mixed. The resulting solution was then slowly added to a solution of 0.2 g (1 mmol) of copper acetate monohydrate in 20 mL of water while stirring. The resulting solution was placed in a thermostat at a constant temperature of 22 °C. After 11 days, green crystals were formed with a yield of 72% (0.31 mg). Elemental analysis for C_34_H_34_Cu_2_O_16_N_2_ (854 g/mol; Calcd: C 47.77%; O 30.0%; H 4.0%; N 3.28%; Found: C 45.83%; O 28.96%; H 4.2%; N 3.32%).

## 3. Results and discussion

### 3.1. FTIR spectra

**Figure 1:**
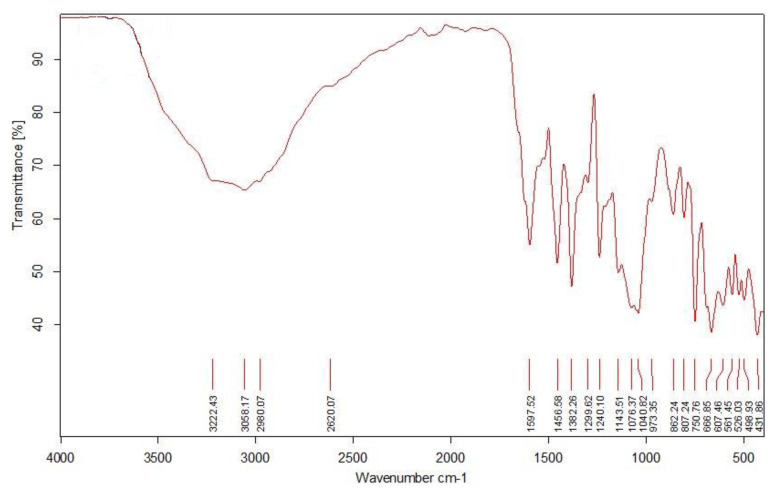
Infrared spectrum of the cocrystal.

In Figure 1, the vibrational spectrum of functional groups and chemical bonds within the composition of the copper coordination compound under the influence of infrared light is illustrated. This analysis revealed valence (ν) and deformation (δ) vibrations present in the functional groups and chemical bonds of the compound.

In the IR spectroscopic range spanning from 3500 cm^−1^ to 2800 cm^−1^, several weak peaks exhibited broadening due to light absorption, indicating average intensity. Notably, the absorption peak at 3222 cm^−1^ corresponded to the valence vibrations of the −OH group, participating in both intermolecular and intramolecular hydrogen bonding.

The asymmetric valence vibrations of the carboxyl group within salicylic acid in the compound displayed strong intensity in the ν_as_ region at 1597 cm^−1^. Additionally, symmetric valence vibrations were observed in the ν_s_ region at 1382 cm^−1^ (ν_as_ – ν_s_ = 215 > 200). These observations indicated that the oxygen atoms of the carboxyl group within the compound were coordinated in a monodentate fashion to the metal. Moreover, strong infrared light absorption was observed at 1456 cm^−1^ in the valence vibrations of the aromatic ring [24,25].

Within the lower range of the IR spectra, absorptions of relatively strong intensity were observed. In this spectral region, various vibrations corresponding to the bonds formed between copper metal and the ligands were identified. Specifically, at 667 cm^−1^, the donor–acceptor bond between water and the metal was observed, while vibrations associated with the bonds between the carboxyl group and the metal were recorded at 607 cm^−1^ and 432 cm^−1^, respectively [26].

### 3.2. UV-Vis spectra

**Figure 2:**
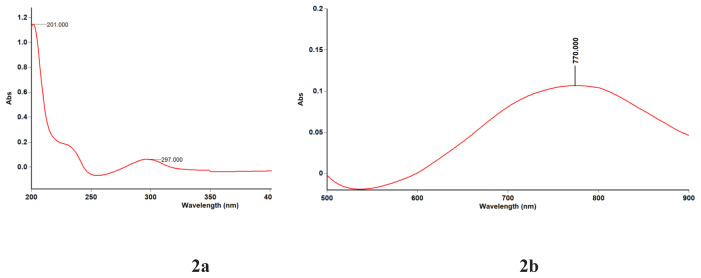
**a, b)** UV spectra of the cocrystal.

Based on the spectra (Figures 2a and 2b), it was determined that the salicylate ligands within the metal complex act as electron donors and are involved in ligand-to-metal charge transfer processes. These transitions occur when electrons move from the ligand orbitals to the high-energy copper d-orbitals (t_2g_→e_g_). Consequently, absorption maxima were observed at longer wavelengths of electromagnetic radiation, specifically at λ_max_ = 770 nm (12,987 cm^−1^) [27].

Furthermore, within the wavelength range of λmax = 297 nm (33,670 cm^−1^) used in the spectrophotometric analysis, metal-to-ligand charge transfer phenomena were observed in the molecule. This process involves transitions to energetically favorable orbitals.

Similarly, within the region of λmax = 201 nm (49,751 cm^−1^), corresponding to shorter wavelengths, n→p* transitions of the carbonyl group within the salicylate ligand were observed.

### 3.3. X-ray crystallography

**Figure 3:**
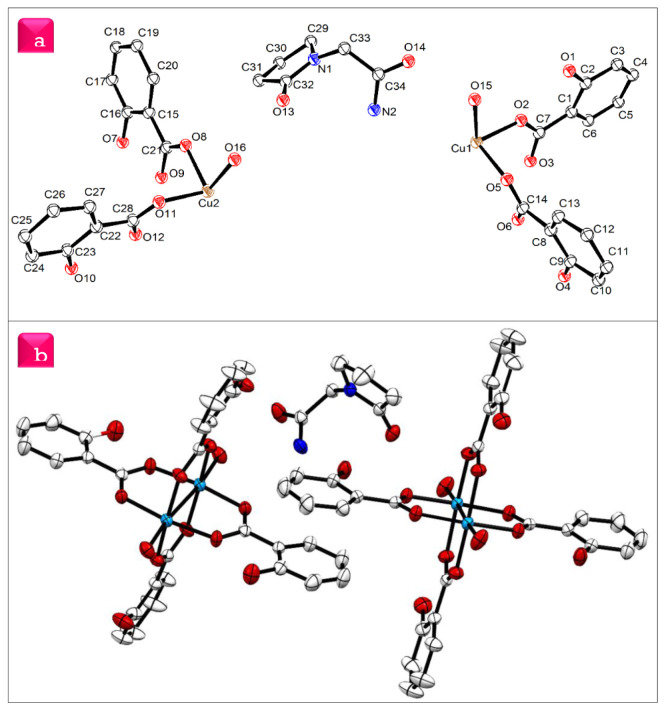
Asymmetry unit **(a)** and ORTEP structure **(b)** of the cocrystal (hydrogen atoms are omitted for clarity and the thermal ellipsoids are drawn at the 30% probability level).

**Figure 4:**
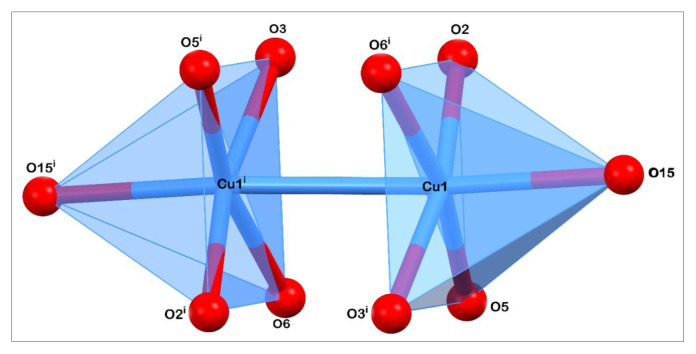
Stereoscopic view of the coordination polyhedron in the cocrystal.

The crystal data and details of data collection and refinement are summarized in Table 1. The asymmetric part of the unit cell consists of three fragments (Figure 3a). They are halves of the two complex molecules and one piracetam (2-(2-oxopyrrolidin-1-yl)acetamide) molecule because complex molecules are located on inversion centers while the uncoordinated piracetam molecule occupies the total position of the unit cell (Figure 3b). Both complex molecules are in the form of a “Chinese lantern,” in which symmetry centers are located between the Cu(II) ions. The dimeric Cu1 and Cu2 ions of independent complex molecules I and II chelate four salicylic acid molecules through oxygen atoms of the carboxylate group and coordinate two water molecules. Oxygen atoms O2, O3, O5, and O6 of molecule I (atoms O8, O9, O11, and O12 for molecule II) are in equatorial positions while oxygen atom O15 (O16 for molecule II) of the water molecule occupies an axial position [28]. Bond lengths of equatorial atoms are in the range of 1.954–1.984 Å, whereas bond distances of axial atoms are 2.120 and 2.106 Å due to Janh–Teller effect. The selected bond lengths, angles, and torsion angles are listed in Tables S1, S2, and S3, respectively. The intermetallic distances of the dimers are 2.643 and 2.621 Å, which are close to the distance in the previously reported copper(II) complex [29]. Orthogonal bond angles vary from 88.09(7)° to 98.74(7)°. Therefore, the coordination polyhedrons of metal atoms are distorted square pyramids. The bond length distortion index is 0.02465 and the effective coordination number is 4.8399. The stereoscopic view of the coordinate polyhedron (Figure 4) and polyhedral geometric parameters are given below (Table 2).

**Figure 5:**
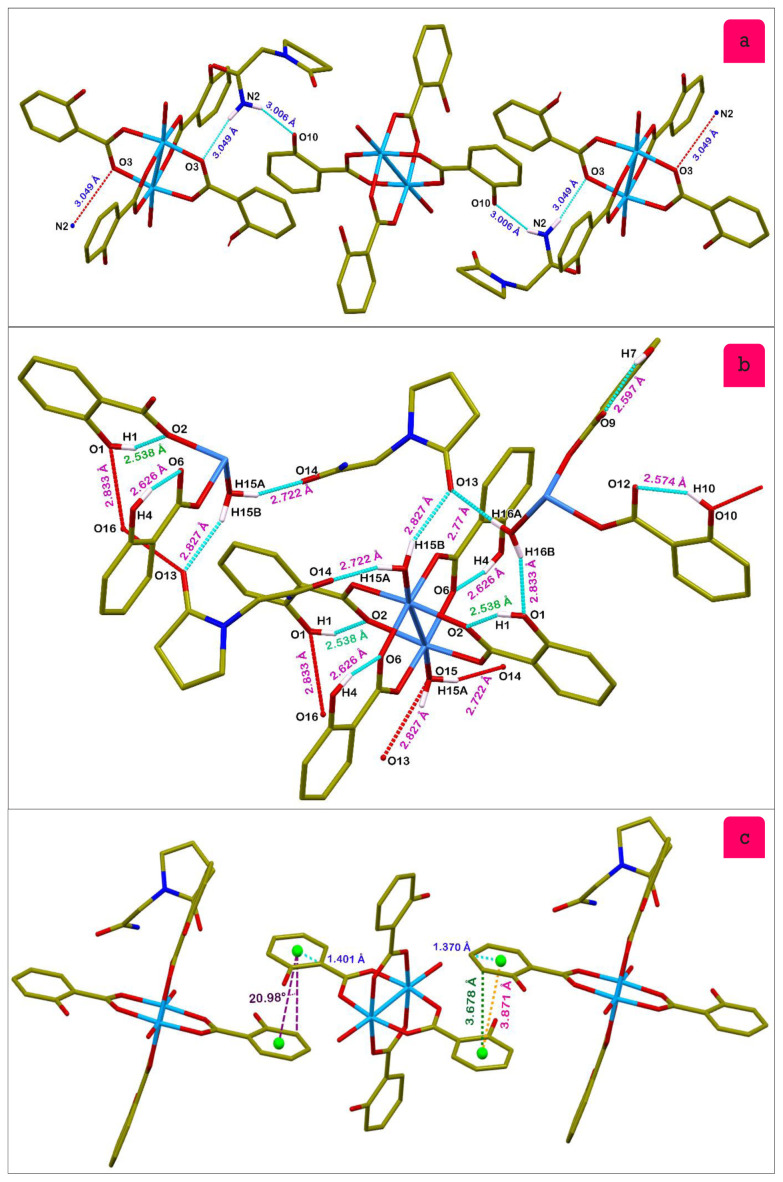
Molecular packing interactions of the O-H•••O **(a, b)** and π•••π **(c)** type (most hydrogens are omitted for clarity).

The hydrogen bonds are listed in Table 3. Hydrogen bonding and weak van der Waals interactions mainly construct a supramolecular architecture. The salicylic acid ligand hydroxyl group makes short intramolecular hydrogen bonds with a distance of 1.822 Å [H(1)•••O(2)]. The 2-(2-oxopyrrolidin-1-yl)acetamide molecule amino group bridged with the two crystallographically independent molecules via N-H•••O hydrogen bonds and the corresponding distances are 3.049(3) Å (N(2)•••O(3)) for the Cu1 molecule and 3.006(3) Å (N(2)•••O(10)) for the Cu2 molecule (Figure 5a).

The Cu1 coordinated water molecules bridged via O-H•••O hydrogen bonds with the nearest two 2-oxopyrrolidin-1-yl)acetamide molecules and the distances are 2.827(3) and 2.722(3) Å for O(15)•••O(13) and O(15)•••O(14), respectively. The Cu2 coordinated water molecules are bridged via O-H•••O hydrogen bonds with 2-oxopyrrolidin-1-yl)acetamide and the Cu1 complex molecule with the distances of 2.770(3) Å (O(16)•••O(13)) and 2.833(3) Å (O(16)•••O(1)) (Figure 5b). The centroid–centroid contact distance is ~3.87 Å with an angle of 20.98°. The stacking arrangement resembles slipped packing with a ring horizontal displacement ranging from 1.370 Å to 1.401 Å and the plane–plane distance is 3.678 Å (Figure 5c).

### 3.4. Hirshfeld surface analysis

Examinations of intermolecular interactions in crystals become quantitative with Hirshfield surface analysis. This method reveals how close neighboring atoms are on both the internal (*d_i_*) and external (*d_e_*) surfaces surrounding each point on the Hirshfield surface. These unique features have proven valuable in understanding the selectivity and specificity of intermolecular forces that influence how molecules pack together.

Generating these surfaces involves dividing the crystal’s space using the Hirshfield ratio. This ratio defines the procrystal, essentially a blueprint for the crystal structure, based on a promolecule with electron density of 0.5. The normalized contact distance, represented by *d_norm_*, is calculated by considering both internal and external perspectives relative to the surface, as the following equation demonstrates:


$$d_{norm}=\frac{d_{i}-r_{i}^{vdW}}{r_{i}^{vdW}}+\frac{d_{e}-r_{e}^{vdW}}{r_{e}^{vdW}}$$

Here, *d_e_* represents the distance from the surface to the closest nucleus outside, while *d_i_* signifies the distance to the nearest one inside. *rvdW* stands for the van der Waals radius of the specific atom.

These distances contribute to the informative *d_norm_* parameter, visualized as a color gradient surface of red to white to blue. Red areas depict intermolecular contacts happening closer than their van der Waals radii, highlighting close interactions. Conversely, blue regions indicate contacts exceeding those radii, signifying a larger gap between molecules. White areas represent the combined van der Waals radii of the involved atoms.

To calculate short-range contacts within the crystal lattice, Crystal Explorer version 21.5 was employed [30]. This software was also used to analyze the crystal structures of Hirshfield surfaces and generate the corresponding two-dimensional (2D) fingerprint plots [31].

In our research, we investigated the arrangement of one molecule of piracetam positioned between two molecules of a binuclear coordination compound consisting of copper metal and salicylic acid. This arrangement played a role in the overall surface formation to some extent. It is worth noting that the piracetam molecule remained neutral, similar to rare cocrystals. This finding aligns with the observations made by Ruzmetov et al. [32].

To comprehensively capture the interactions within the molecular crystals, we utilized the entire compound molecule for the calculation of the Hirshfeld surface. The primary objective was to provide a comprehensive representation of the molecular interactions. The Hirshfeld surface of the compound was mapped by calculating the normalized contact distance *d_norm_* and the resulting surface was visualized using indicator colors such as red, white, and blue. The obtained results revealed a range of recorded interactions in molecular crystals, spanning atomic sizes from −0.6259 (indicated by red) to 2.0808 (indicated by blue).

**Figure 6:**
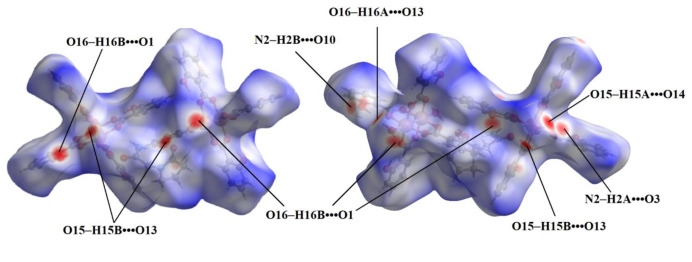
The Hirshfeld surface of the cocrystal.

The total Hirshfeld surface area was determined to be 1665.66 Å^2^, with a specific surface area of 1132.36 Å^2^. Notably, the interactions within the molecular crystals prominently involved oxygen atoms, as illustrated in Figure 6.

**Figure 7:**
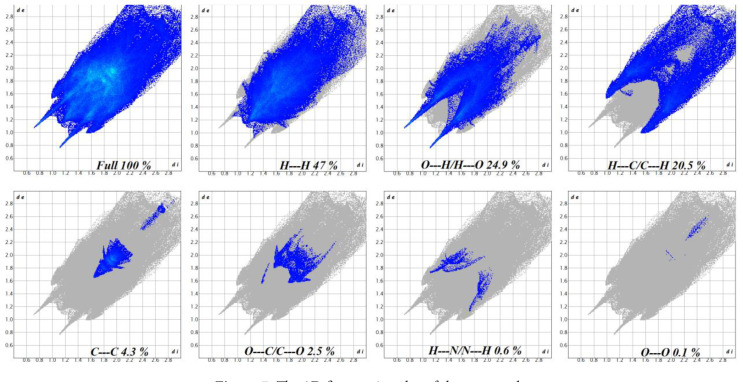
The 2D fingerprint plot of the cocrystal.

Analysis of the 2D fingerprint plot of the compound’s Hirshfeld surface (Figure 7) revealed the dominance of hydrogen–hydrogen (H•••H) interactions, contributing approximately 47% of the overall surface. Notably, these interactions play a crucial role in crystal packing. Additionally, significant contributions are observed from O•••H/H•••O interactions (24.9%), primarily involving oxygen atoms acting as hydrogen bond acceptors and donors. These interactions further enhance the stability of the crystal structure. Additionally, hydrogen–carbon interactions (H•••C/C•••H) account for 20.5% of the surface formation. Interactions involving carbon atoms (C•••C) and oxygen–carbon contacts (O•••C/C•••O) play a minor role (4.5% and 2.5%, respectively). Interestingly, the fingerprint plot reveals minimal contributions from hydrogen–nitrogen interactions (H•••N/N•••H, 0.6%) and oxygen–oxygen interactions (O•••O, 0.1%).

**Figure 8:**
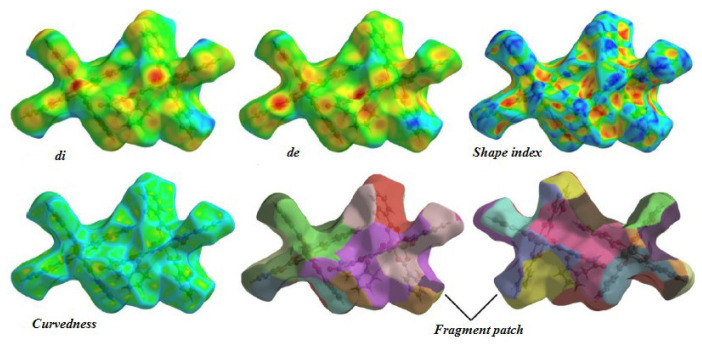
Important parameters of the Hirshfeld surfaces: di, de, shape index, curvature, and fragment patch.

The analysis of the Hirshfeld surfaces of a chemical compound incorporated several indicators, namely di, de, shape index, curvedness, and fragment patches (Figure 8). It is noteworthy that di ranged from 0.7246 to 3.2227, while de ranged from 0.7668 to 3.3372. The observation that di < de suggests a higher electron density within the Hirshfeld surface of the crystal.

Additionally, the shape index exhibited values ranging from −0.9995 to 0.9977 in terms of atomic dimensions. It is important to highlight that the shape index revealed a distance of 2.074 Å between the C3 and C23 atoms, indicating that the aromatic ring in the molecule is positioned opposite the generalized p orbital. This arrangement leads to π–π coverage between the opposing C atoms.

The curvedness values ranged between −4.104 and 0.866. A high negative value denotes significant concavity and nonconvexity on the Hirshfeld surface, whereas a positive value signifies a moderately convex region. In other words, a positive value indicates that the surface protrudes slightly outward.

### 3.5. Molecular docking

**Figure 9:**
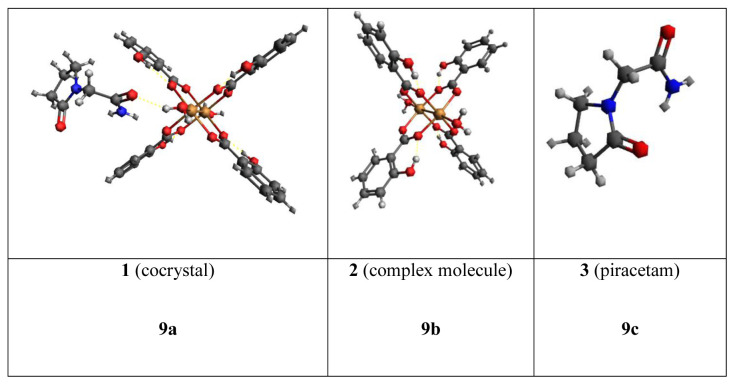
**a–c)** Structures of the compounds for molecular docking studies.

Molecular docking studies were conducted to assess the potential antifungal, antibacterial, and antiviral activities of the investigated compounds in interaction with proteins of microorganisms and viruses [33,34]. The 3D geometry of the cocrystal was taken from the cif-file of the compound, whereas the 3D geometry of the piracetam was prepared using Avogadro software [35]. The structures of proteins tLeuRS (PDB ID: 2V0C), *E. coli* gyrase B (PDB ID: 6F86), *C. albicans* (PDB ID: 2QZX), and the main protease (Mpro) of SARS-CoV-19 (PDB ID: 6LU7) were obtained from the PDB database [36] as pdb files and their structures were cleaned of additional molecules using the Biovia DS visualizer program (Dassault Systèmes, Vélizy-Villacoublay, France). The structures of compounds **1**–**3** (Figures 9a–9c) and cleaned proteins were converted to pdbqt files in the AutoDockTools-1.5.7 program package and these pdbqt files were used for molecular docking studies with the AutoDock 4.2 program [37]. Previously used active sites (x-, y-, and z-centers of the active sites) of proteins [38,39] were selected for molecular docking studies in this work. The molecular docking results were visualized with the Biovia DS visualizer program [40].

**Figure 10:**
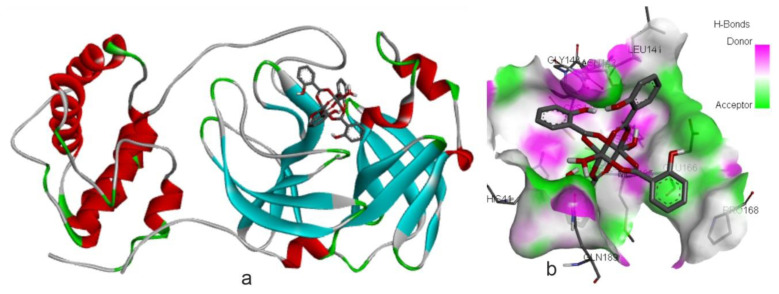
Compound **2** in the active site of protein 6LU7 **(a)** and its interactions by H-bonds **(b)**.

Compound **1** was designed as a hydrogen bonding system and its structural integrity was maintained throughout the molecular docking studies. In all cases, the binding energy of compound **1** was found to be optimal compared to that of compounds **2** and **3** (Table 4). The highest binding energy for compound **1** was observed while determining its antiviral activity, with a value of BE = −11.40 kcal/mol. Compound **2** also exhibited relatively good results against SARS-CoV-2 Mpro, with binding energy of −10.34 kcal/mol, indicating its potential efficacy (Table 3). The compounds fit well in the active site of the protein. In Figure 10a and Figure 10b, the localization of **2** in the active site is illustrated, along with its interaction with amino acid residues through H-bonds.

**Figure 11a:**
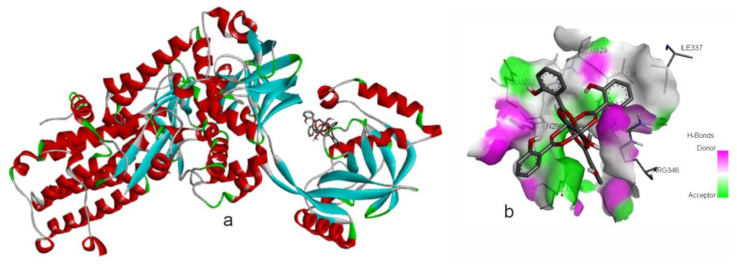
Compound **2** in the active site of protein 2V0C **(a)** and its interactions with H-bonds **(b)**.

The receptor protein tLeuRS (PDB ID: 2V0C) was previously used by Zhang and colleagues [41,42] in antifungal studies of some selected compounds. Interactions between compounds **1**–**3** and this protein were evaluated to assess their potential antifungal activity. The binding energy of compound **1** (−9.26 kcal/mol) suggests potential for exhibiting antifungal activity. Hydroxyl groups and water molecules of compound **2** can act as H-bond donors and they may interact with H-bond acceptors, such as carbonyl groups (C=O) found in amino acid residues (Figures 11a and 11b).

## 4. Conclusion

The cocrystal of the Cu(II) complex of salicylic acid and piracetam has been synthesized and structurally characterized. The binuclear Cu(II) complex of salicylic acid (metal complex), which adopts a “Chinese lantern” structure, interacts with uncoordinated piracetam through a complicated system of hydrogen bonds. In silico (molecular docking) studies of the cocrystal, the metal complex, and piracetam’s biological actions were conducted using AutoDock 4.2. These research results indicated that the metal complex exhibits enhanced antifungal, antibacterial, and antiviral activities. Although piracetam is practically inactive on its own, it enhances all tested bioactivities when the cocrystal is formed with the Cu(II) complex of salicylic acid, improving the binding energy from −10.34 to −11.40 kcal/mol. Therefore, the formation of cocrystals based on metal complexes and inactive organic compounds may hold promise in drug design.

## Data availability

Data availability is not applicable to this study.

## Supplementary Information

**Table S1:** Selected bond lengths [Å] for the cocrystal.

Bond	Length [Å]	Bond	Length [Å]	Bond	Length [Å]
Cu2-Cu2	2.6208(6)	N1-C32	1.335(3)	C1-C6	1.394(4)
Cu2-O12	1.9845(16)	N1-C33	1.446(3)	C34-C33	1.516(3)
Cu2-O11	1.9659(16)	N1-C29	1.461(3)	C2-C3	1.385(4)
Cu2-O9	1.9744(17)	O1-C2	1.356(3)	C16-C17	1.390(4)
Cu2-O8	1.9539(17)	O10-C23	1.349(3)	C23-C24	1.397(4)
Cu2-O16	2.1062(17)	O7-C16	1.343(4)	C9-C10	1.391(4)
Cu1-Cu1	2.6428(6)	O14-C34	1.225(3)	C31-C30	1.496(5)
Cu1-O3	1.9682(16)	O4-C9	1.363(4)	C6-C5	1.382(4)
Cu1-O2	1.9722(16)	N2-C34	1.319(3)	C20-C19	1.373(4)
Cu1-O6	1.9719(17)	C7-C1	1.477(3)	C27-C26	1.381(4)
Cu1-O15	2.1203(17)	C28-C22	1.478(3)	C13-C12	1.384(4)
Cu1-O5	1.9536(16)	C8-C14	1.480(3)	C29-C30	1.519(5)
O12-C28	1.274(3)	C8-C9	1.390(4)	C10-C11	1.359(5)
O3-C7	1.260(3)	C8-C13	1.373(4)	C24-C25	1.369(5)
O11-C28	1.261(3)	C21-C15	1.482(3)	C3-C4	1.373(5)
O2-C7	1.272(3)	C22-C23	1.407(3)	C26-C25	1.389(5)
O6-C14	1.277(3)	C22-C27	1.390(3)	C5-C4	1.374(5)
O9-C21	1.271(3)	C32-C31	1.499(4)	C17-C18	1.349(5)
O8-C21	1.260(3)	C15-C16	1.405(4)	C18-C19	1.393(5)
O5-C14	1.254(3)	C15-C20	1.388(4)	C11-C12	1.364(5)
O13-C32	1.235(3)	C1-C2	1.400(4)		

Symmetry transformations used to generate equivalent atoms.

**Table S2:** Selected bond angles [°] for the cocrystal.

Bond	Angle [°]	Bond	Angle [°]	Bond	Angle [°]
O12-Cu2-Cu2	84.31(5)	C21-O9-Cu2	123.11(16)	N2-C34-C33	117.8(2)
O12-Cu2-O16	92.18(7)	C21-O8-Cu2	123.64(16)	N1-C33-C34	116.4(2)
O11-Cu2-Cu2	84.51(5)	C14-O5-Cu1	127.46(16)	O1-C2-C1	122.6(2)
O11-Cu2-O12	168.79(7)	C32-N1-C33	124.6(2)	O1-C2-C3	117.3(3)
O11-Cu2-O9	90.70(7)	C32-N1-C29	113.5(2)	C3-C2-C1	120.0(3)
O11-Cu2-O16	99.00(7)	C33-N1-C29	121.6(2)	O7-C16-C15	123.2(2)
O9-Cu2-Cu2	84.08(5)	O3-C7-O2	123.6(2)	O7-C16-C17	117.7(3)
O9-Cu2-O12	88.93(7)	O3-C7-C1	119.3(2)	C17-C16-C15	119.0(3)
O9-Cu2-O16	96.13(9)	O2-C7-C1	117.1(2)	O10-C23-C22	122.6(2)
O8-Cu2-Cu2	84.71(5)	O12-C28-C22	118.1(2)	O10-C23-C24	118.0(2)
O8-Cu2-O12	89.29(7)	O11-C28-O12	123.8(2)	C24-C23-C22	119.4(3)
O8-Cu2-O11	88.91(8)	O11-C28-C22	118.1(2)	O4-C9-C8	123.3(2)
O8-Cu2-O9	168.76(7)	C9-C8-C14	122.8(2)	O4-C9-C10	117.2(3)
O8-Cu2-O16	95.03(9)	C13-C8-C14	118.4(2)	C8-C9-C10	119.5(3)
O16-Cu2-Cu2	176.49(6)	C13-C8-C9	118.7(2)	C30-C31-C32	104.3(2)
O3-Cu1-Cu1	88.46(5)	O6-C14-C8	118.2(2)	C5-C6-C1	120.1(3)
O3-Cu1-O2	167.95(7)	O5-C14-O6	123.9(2)	C19-C20-C15	121.3(3)
O3-Cu1-O6	88.09(7)	O5-C14-C8	117.9(2)	C26-C27-C22	121.5(3)
O3-Cu1-O15	98.74(7)	O9-C21-C15	118.0(2)	C8-C13-C12	121.6(3)
O2-Cu1-Cu1	79.51(5)	O8-C21-O9	124.3(2)	N1-C29-C30	103.0(2)
O2-Cu1-O15	93.31(7)	O8-C21-C15	117.7(2)	C11-C10-C9	120.1(3)
O6-Cu1-Cu1	86.49(5)	C23-C22-C28	121.4(2)	C25-C24-C23	120.4(3)
O6-Cu1-O2	90.45(8)	C27-C22-C28	119.8(2)	C4-C3-C2	119.5(3)
O6-Cu1-O15	95.99(7)	C27-C22-C23	118.7(2)	C27-C26-C25	119.0(3)
O15-Cu1-Cu1	172.44(6)	O13-C32-N1	124.2(2)	C4-C5-C6	119.7(3)
O5-Cu1-Cu1	81.49(5)	O13-C32-C31	126.9(2)	C18-C17-C16	121.0(3)
O5-Cu1-O3	89.64(8)	N1-C32-C31	108.9(2)	C24-C25-C26	120.9(3)
O5-Cu1-O2	89.28(8)	C16-C15-C21	121.8(2)	C3-C4-C5	121.4(3)
O5-Cu1-O6	167.81(7)	C20-C15-C21	119.3(2)	C17-C18-C19	120.8(3)
O5-Cu1-O15	96.18(7)	C20-C15-C16	118.8(2)	C20-C19-C18	119.0(3)
C28-O12-Cu2	123.16(15)	C2-C1-C7	121.0(2)	C10-C11-C12	121.3(3)
C7-O3-Cu1	119.15(15)	C6-C1-C7	119.8(2)	C11-C12-C13	118.7(3)
C28-O11-Cu2	124.22(15)	C6-C1-C2	119.2(2)	C31-C30-C29	105.8(3)
C7-O2-Cu1	129.26(16)	O14-C34-N2	124.1(2)		
C14-O6-Cu1	120.22(16)	O14-C34-C33	118.1(2)		

Symmetry transformations used to generate equivalent atoms.

**Table S3:** Torsion angles [°] for the cocrystal.

Dihedral bond	Angle [°]	Dihedral bond	Angle [°]
Cu2-O12-C28-O11	−2.1(3)	C14-C8-C9-O4	−3.1(4)
Cu2-O12-C28-C22	178.03(14)	C14-C8-C9-C10	176.1(3)
Cu2-O11-C28-O12	1.1(3)	C14-C8-C13-C12	−176.4(3)
Cu2-O11-C28-C22	−179.10(14)	C21-C15-C16-O7	−2.0(4)
Cu2-O9-C21-O8	4.5(3)	C21-C15-C16-C17	178.9(3)
Cu2-O9-C21-C15	−174.38(15)	C21-C15-C20-C19	−179.0(3)
Cu2-O8-C21-O9	−5.5(3)	C22-C23-C24-C25	2.0(4)
Cu2-O8-C21-C15	173.39(15)	C22-C27-C26-C25	2.5(5)
Cu1-O3-C7-O2	−0.9(3)	C32-N1-C33-C34	93.0(3)
Cu1-O3-C7-C1	178.50(15)	C32-N1-C29-C30	−11.5(4)
Cu1-O2-C7-O3	2.1(4)	C32-C31-C30-C29	−20.5(4)
Cu1-O2-C7-C1	−177.34(15)	C15-C16-C17-C18	0.1(5)
Cu1-O6-C14-O5	5.6(3)	C15-C20-C19-C18	0.1(6)
Cu1-O6-C14-C8	−172.56(16)	C1-C2-C3-C4	1.3(4)
Cu1-O5-C14-O6	−8.9(4)	C1-C6-C5-C4	0.5(5)
Cu1-O5-C14-C8	169.26(16)	C33-N1-C32-O13	−7.3(4)
O12-C28-C22-C23	6.7(3)	C33-N1-C32-C31	171.6(2)
O12-C28-C22-C27	−171.6(2)	C33-N1-C29-C30	175.2(3)
O3-C7-C1-C2	−170.2(2)	C2-C1-C6-C5	1.2(4)
O3-C7-C1-C6	9.6(3)	C2-C3-C4-C5	0.4(5)
O11-C28-C22-C23	−173.2(2)	C16-C15-C20-C19	−0.4(5)
O11-C28-C22-C27	8.5(3)	C16-C17-C18-C19	−0.4(7)
O2-C7-C1-C2	9.3(3)	C23-C22-C27-C26	−0.3(4)
O2-C7-C1-C6	−171.0(2)	C23-C24-C25-C26	0.2(5)
O9-C21-C15-C16	0.0(4)	C9-C8-C14-O6	−0.8(4)
O9-C21-C15-C20	178.5(2)	C9-C8-C14-O5	−179.1(2)
O8-C21-C15-C16	−179.0(2)	C9-C8-C13-C12	1.6(5)
O8-C21-C15-C20	−0.5(4)	C9-C10-C11-C12	−2.0(7)
O13-C32-C31-C30	−167.1(3)	C6-C1-C2-O1	176.6(2)
N1-C32-C31-C30	14.0(4)	C6-C1-C2-C3	−2.1(4)
N1-C29-C30-C31	19.5(4)	C6-C5-C4-C3	−1.3(6)
O1-C2-C3-C4	−177.4(3)	C20-C15-C16-O7	179.5(3)
O10-C23-C24-C25	−178.0(3)	C20-C15-C16-C17	0.4(4)
O7-C16-C17-C18	−179.1(3)	C27-C22-C23-O10	178.1(2)
O14-C34-C33-N1	160.0(2)	C27-C22-C23-C24	−1.9(4)
O4-C9-C10-C11	−178.7(3)	C27-C26-C25-C24	−2.5(6)
N2-C34-C33-N1	−21.9(4)	C13-C8-C14-O6	177.2(2)
C7-C1-C2-O1	−3.6(4)	C13-C8-C14-O5	−1.1(4)
C7-C1-C2-C3	177.7(2)	C13-C8-C9-O4	179.0(3)
C7-C1-C6-C5	−178.6(3)	C13-C8-C9-C10	−1.8(4)
C28-C22-C23-O10	−0.3(4)	C29-N1-C32-O13	179.6(3)
C28-C22-C23-C24	179.7(2)	C29-N1-C32-C31	−1.4(3)
C28-C22-C27-C26	178.0(3)	C29-N1-C33-C34	−94.5(3)
C8-C9-C10-C11	2.0(5)	C10-C11-C12-C13	1.8(7)
C8-C13-C12-C11	−1.6(6)	C17-C18-C19-C20	0.4(7)

Symmetry transformations used to generate equivalent atoms.

## Figures and Tables

**Table 1:** Crystal data and structural refinement of the cocrystal.

CCDC	2344454
Empirical formula	C_8.50_H_8.50_Cu_0.50_ N_0.50_O_4_
Moiety formula	0.25(C_28_H_24_Cu_2_O_14_),0.25(C_6_H_10_N_2_O_2_)
Formula weight	213.43
Temperature (K)	293(2)
Radiation type	CuKα (1.54184 Å)
Crystal system	Triclinic
Space group	P-1
a, b, c (Å)	10.4249(1), 10.5644(1), 17.3440(2)
α, β, γ (°)	98.995(1), 97.220(1), 91.423(1)
Volume (Å^3^)	1869.76(3)
Z	8
Density (calculated) (Mg/m^3^)	1.516
Absorption coefficient (mm^−1^)	2.057
F(000)	876
Crystal size (mm^3^)	0.140 x 0.120 x 0.110
Theta range for data collection	2.5970 to 71.4480°
Index ranges	−12 ≤ h ≤ 11, −12 ≤ k ≤ 12, −20 ≤ l ≤ 21
Reflections collected	7172
Independent reflections	5915 [R(int) = 0.0303]
Data/restraints/parameters	7172 / 1 / 496
Goodness-of-fit on F^2^	1.098
Absorption correction	multiscan
Max. and min. transmission	0.762 and 1.000
Refinement method	Full-matrix least-squares on F^2^
Final R indices [I>2sigma(I)]	R1 = 0.0350, wR2 = 0.0938
R indices (all data)	R1 = 0.0432, wR2 = 0.1014
Extinction coefficient	0.00080(9)
Largest diff. peak and hole (e.Å^−3^)	0.349 and −0.351

**Table 2:** Polyhedral geometric parameters for Cu(1) and Cu(2).

Bond	Distances (Å)	Bond	Distances (Å)
Cu(1)-O(6)	1.9719(19)	Cu(2)-O(8)	1.9539(18)
Cu(1)-O(15)	2.1204(17)	Cu(2)-O(16)	2.1060(19)
Cu(1)-O(2)	1.9723(19)	Cu(2)-O(11)	1.9660(19)
Cu(1)-O(3)	1.9682(18)	Cu(2)-O(12)	1.9845(17)
Cu(1)-O(5)	1.9535(18)	Cu(2)-O(9)	1.9745(17)
Average bond length (Å)	**1.9973**	**1.9970**	
Polyhedral volume (Å^3^)	**5.9283**	**5.8781**	
Distortion index (bond length)	**0.02465**	**0.02185**	
Effective coordination number	**4.8399**	**4.8687**	

**Table 3:** Geometric details of hydrogen bonding [Å and °] in the cocrystal.

D-H•••A	d(D-H)	d(H•••A)	d(D•••A)	<(DHA)
N(2)-H(2A)•••O(3)	0.86	2.201	3.049(3)	169
N(2)-H(2B)•••O(10)	0.86	2.188	3.006(3)	158.9
O(1)-H(1)•••O(2)	0.82	1.822	2.538(3)	145.1
O(4)-H(4)•••O(6)	0.82	1.920	2.626(3)	143.7
O(7)-H(7)•••O(9)	0.82	1.897	2.597(3)	142.6
O(10)-H(10)•••O(12)	0.82	1.861	2.574(2)	144.7
O(15)-H(15A)•••O(14)	0.853	1.940	2.722(3)	152
O(15)-H(15B)•••O(13)	0.853	2.019	2.827(3)	157.7
O(16)-H(16A)•••O(13)	0.85	1.929	2.770(3)	169.9
O(16)-H(16B)•••O(1)	0.85	2.012	2.833(3)	162

Symmetry transformations used to generate equivalent atoms: 1-x,-y,1-z, 1-x,1-y,-z, 1-x,1-y,1-z

**Table 4:** Binding energies of the compounds.

Compound	Receptor (PDB ID)	Binding energy (BE), kcal/mol	H-bond contacting residues
1	tLeuRS (2V0C)	−9.26	THR247, THR248, ARG249, GLN296, LEU329, ARG346
2	tLeuRS (2V0C)	−8.87	GLU292, ARG295, GLN296, TYR327, LEU329
3	tLeuRS (2V0C)	−5.57	LYS290, GLU294, ARG295, ARG300, THR303
1	E.Coli (6F86)	−8.41	ASN46, VAL120
2	E.Coli (6F86)	−8.30	GLU42, ASP49, ILE94, SER121
3	E.Coli (6F86)	−4.76	ASP73, THR165, VAL167
1	C.Albicans (2QZX)	−8.89	LYS83, TYR84, GLY85, ASP303
2	C.Albicans (2QZX)	−8.37	ASP86, ARG299
3	C.Albicans (2QZX)	−5.28	THR33, PHE128, LEU194
1	Mpro (6LU7)	−11.40	ASN142, GLY143, HIS164, GLU166
2	Mpro (6LU7)	−10.34	LEU141, ASN142, SER144, CYS145, HIS164, GLN189
3	Mpro (6LU7)	−4.63	GLY143, SER144, HIS163
